# Burdens of Gastroesophageal Reflux Disease in China: Findings from the 2021 GBD Study

**DOI:** 10.1371/journal.pone.0334719

**Published:** 2025-10-17

**Authors:** Zhiqiang Liang, Zehui Hou, Zhuomin Yu, Bing Zeng, Fang Li, Jingjing Wu, Yingru Li, Zhipeng Jiang

**Affiliations:** 1 Department of General Surgery, Hernia and Abdominal Wall Surgery, Guangdong Provincial Key Laboratory of Colorectal and Pelvic Floor Diseases, Biomedical Innovation Center, The Sixth Affiliated Hospital, Sun Yat-sen University, Guangzhou, Guangdong Province, China; 2 Division of Gastrointestinal Surgery, Department of General Surgery, Shenzhen People’s Hospital (The Second Clinical Medical College, Jinan University, Shenzhen, Guangdong Province, China; 3 The First Affiliated Hospital, Southern University of Science and Technology), Shenzhen, Guangdong Province, China; University of Bari: Universita degli Studi di Bari Aldo Moro, ITALY

## Abstract

As a major global health concern, Gastroesophageal Reflux Disease (GERD) has garnered widespread attention; However, to date, the prevalence of GERD across different ethnic groups in China has not been comprehensively examined by any large-scale epidemiological studies or meta-analyses. Against this backdrop, the present study draws on data from the Global Burden of Disease (GBD) Study to investigate the burden of GERD in China. Data from the 2021 GBD Study were utilized to assess the burden of GERD in China, focusing on key indicators such as prevalence, incidence, Years of Life Lived with Disability (YLDs), Age-Standardized Rates (ASR). Health inequality analyses were used to measure the inequality in the distribution of GERD across countries based on the Socio-Demographic Index (SDI), as well as to determine whether such inequality exists in China. Frontier analysis identified top performers in healthcare systems across different countries when examining the burden of GERD, followed by a comparison of differences in GERD burden between China and these top-performing countries. The trends of GERD from 2022 to 2044 were projected using the Nordpred and ARIMA model. Between 1990 and 2021, the prevalence of GERD in China experienced a notable rise of 60.62%. Correspondingly, the ASR also increased significantly. A similar pattern was Observed in incidence and YLDs, with number and ASR rising. A frontier analysis revealed that China had relatively low prevalence and incidence rates but a moderate-to-high level of SDI. Health inequality analysis revealed both absolute and relative inequalities related to SDI, with a noticeable increase in the inequality of the age-standardized GERD burden from 1990 to 2021. a significantly greater inequality in GERD burden was observed in China compared to other countries, suggesting a disparity in GERD distribution relative to SDI. Projections of GERD in China extending from 1990 to 2044 indicate a steady increase in the combined numbers and ASR of prevalence, incidence, and YLDs. The disease burden of GERD in China has risen steadily over the past three decades, with marked increases in the ASRs of its prevalence, incidence, and YLDs. Given the growing number of individuals affected by GERD, adapting healthcare systems to address the escalating demand for related services and treatments has become an urgent necessity.

## Introduction

Gastroesophageal Reflux Disease (GERD) is a chronic disorder characterized by the recurrent and ongoing regurgitation of stomach contents into the esophagus. This process can induce a spectrum of both symptomatic and histological alterations in the affected tissues [1]. GERD has emerged as a significant health concern, affecting a considerable proportion of the population, with prevalence rates reaching up to 20% in some Western countries. Moreover, the incidence of GERD is increasing globally, posing a growing public health challenge [2]. The disease is typically marked by frequent symptoms such as heartburn and acid reflux. Additionally, the backflow of gastric contents can cause mucosal injury, primarily in the distal esophagus, which is a hallmark of the condition [1,3]. GERD can result in various histological changes within the esophageal mucosa, including non-erosive reflux disease, reflux esophagitis, and Barrett’s esophagus, all of which contribute to the complexity and potential complications of the disease [4]. In addition to these typical esophageal symptoms, GERD can also lead to a range of extraesophageal manifestations, such as respiratory issues like asthma, chronic cough, sore throat, throat clearing, and even unexplained chest pain, which can complicate diagnosis and management [1]. Furthermore, GERD has been associated with other significant health conditions, including sleep disturbances and an increased risk of depression, both of which contribute to the overall disease burden [5,6]. The recurrent nature of GERD and its potential to cause severe complications, such as esophageal inflammation, strictures, ulcers, perforation, metaplasia, and the development of esophageal adenocarcinoma, significantly impacts patients’ physical and mental well-being [7]. This ongoing burden not only diminishes quality of life but also places a substantial economic strain on healthcare systems, families, and society at large, due to the high costs of treatment and the significant consumption of medical resources [8]. The complex interplay of factors influencing GERD, ranging from individual health behaviors to broader socio-economic determinants, highlights the need for comprehensive management strategies to address both the immediate and long-term impacts of the disease [4].

In-depth investigation and analysis of the epidemiological trends of GERD are particularly important for policy formulation and improvement. Although numerous systematic reviews and cross-sectional surveys have been published to evaluate the prevalence and incidence of GERD globally and in specific regions or countries [9,10], the existing data is still inadequate for a comprehensive understanding. A systematic analysis of the global GERD burden was carried out by the Global Burden of Disease (GBD) 2019 GERD collaborators [11,12]. However, those studies on GERD have prioritized exploring global or continental patterns in disease burden distribution. This analytical framework does not involve in-depth analysis of the burden characteristics specific to individual countries. Thus, previous global burden studies on GERD are unfavorable for informing policy formulation within a specific country—for instance, China. This is because the burden of GERD exhibits inconsistencies across different regions and time periods, as it is influenced by a range of factors, including regional disparities, temporal variations, population demographics, survey methodologies, and the diagnostic criteria employed. Given China’s vast geographical area and ethnically diverse population, it is anticipated that the burden pattern of GERD in China differs from the global pattern [13]. Most existing studies on GERD in China have been limited to specific cities or small regions, often with a narrow scope. To date, no large-scale, ethnicity-inclusive epidemiological study or meta-analysis has been conducted to comprehensively cover the GERD burden across the entire Chinese population.

During the last 30 years China experienced major social and population changes which show the reason the China need planned data tracking for GERD status. Our health programs need these studies to battle growing medical issues linked to GERD. GBD 2021 represents the largest global study ever done to research disease patterns across 371 conditions throughout different populations [14,15]. Based on GBD 2021 Study, our research examined the demographic and disease factors that influenced the rise and spread of GERD across China from 1990 to 2021, and projected the disease trends up to 2044. These epidemiological trends were analyzed to support health policy formulation in China and address the need for foresight in chronic disease control as required by long-term initiatives such as the “Healthy China 2030” program. The data enables us to find the critical areas for GERD prevention and helps create strong health policies and allocate resources where they matter most.

## Materials and methods

### Data sources

The GERD data covering the period from 1990 to 2021 were obtained through the Global Health Data Exchange (GHDx) query tool (https://ghdx.healthdata.org/gbd-2021). This study utilized a comprehensive dataset from the GBD Study 2021, which includes data on Age-Standardized Rates (ASRs), prevalence, incidence, and Years of Life Lived with Disability (YLDs) for GERD across 204 countries. The data were disaggregated by sex (both sexes combined, male, and female) and categorized into 19 distinct age groups, spanning from less than five years to ninety-five years or older, with five-year intervals. The research team divided the data into five identification groups using Socio-Demographic Index (SDI) which ranges between 0 and 1. A higher SDI score shows how well developed a specific region’s economy has become [16]. These regions were grouped into five SDI categories: low (<0.454743), low-middle (0.454743–0.607679), middle (0.607679–0.689504), high-middle (0.689504–0.805129), and high (>0.805129). In addition to SDI classifications, the regions were also separated into four income-based categories, as defined by the World Bank: our SDI ranking divides nations into high, upper-middle, middle, lower-middle and low income groups.

### Statistical analysis

The analysis of our dataset commenced with an examination of its structure, focusing on key metrics such as ASRs, prevalence, incidence, and YLDs for GERD in China. We calculated counts and rates for these metrics and examined the variations over the period from 1990 to 2021. To assess the relative changes, we applied the following formula:


Relative change (%)=[(Value in 2021−Value in 1990)/Value in 1990]×100%.

Our method worked equally for both case number data and ASRs expressed per 100,000 population. A sophisticated statistical method helped us track GERD trends based on Annual Percentage Change (APC) results plus Estimated Annual Percentage Changes (EAPC) and Average Annual Percentage Change (AAPC) outcomes [16–18]. APC represents the yearly variations, while the EAPC was calculated to estimate the long-term trend in ASRs of GERD burden. The EAPC was derived through a regression model by fitting the natural logarithm of ASR with the calendar year [17], expressed as y = α + βx + ε, where y represents the logarithm of the rate, x is the calendar year, and ε is the error term. AAPC measures the typical movement of rates within specific time periods. Joinpoint regression methods help spot how data rates change through time periods to find real pattern shifts from ordinary variations. The joinpoint regression model is a collection of linear statistical models that were used to evaluate the trends in disease burdens attributable to GERD across time [19]. This model’s calculating approach is to estimate the changing rule of illness rates using the least square method, avoiding the non-objectivity of typical trend analyses based on linear trends. Calculating the square sum of the residual error between the estimated and actual values yields the turning point of the shifting trend [19]. When analyzed in conjunction with Joinpoint Regression, the AAPC captures the segmented trends derived from joinpoint regression, facilitating the identification of differences across distinct time periods. Joinpoint Trend Analysis Software version 4.7.0.0 analyzed GERD case data from 1990 through 2021 to calculate the AAPC.

Frontier analysis helped compare which areas performed most effectively against successful countries when examining GERD burden [20]. The core of frontier analysis lies in first defining an “optimal standard” and then measuring the gap between each decision-making unit and this standard. In the present study, frontier analyses were constructed based on the SDI and ASRs of incidence, prevalence, and YLDs. The frontier line delineates the countries and territories that achieve the lowest incidence, prevalence, and YLDs rates (i.e., optimal performers) given their specific SDI levels [20]. The distance from the frontier is termed the “effective difference,” which represents the gap between a country’s observed values and its potentially achievable values [20]. This gap could potentially be reduced or eliminated by leveraging the sociodemographic resources available to the respective country or territory. Our system picks top performers in healthcare from various countries to help other regions set their improvement targets. Our research technique shows the differences between China’s performance and other countries’ levels. To quantify these differences, the ‘effective difference’ was calculated for each country and region, reflecting the gap between the current GERD burden and its potential, adjusted for SDI.

The inequality analysis used both Slope Index of Inequality(SII) and Concentration Index (CI) methods to measure the distributed inequality of GERD based on SDI in each country. The SII is a quantitative indicator that measures absolute differences in health status by socioeconomic status (e.g., income, SDI) through regression analysis [21]. SDI ranked countries, and SII was calculated by robust regression of the midpoint of the cumulative population distribution. The Concentration Index measures relative inequality by comparing the cumulative distribution of prevalence to the population ranked by SDI, highlighting areas with significant health disparities [21]. CI is calculated by integrating the area under the Lorenz curve, reflecting the cumulative distribution of disease prevalence against SDI [21]. A regression model helped us determine the SII by showing how each country’s GERD rates change from their position on the sociodemographic development scale [22]. We measured GERD inequality by finding the middle point between all population SDI rankings. This measurement system created precise results for each population sub-group. We built a Lorenz curve to show both SDI-ranked population groups and disease occurrence rates to determine coverage statistics. Our numerical calculation of area under the Lorenz curve generated the precise CI that shows how diseases spread differently across different population groups [23].

The Bayesian Age-Period-Cohort (BAPC) model has always been employed to analyze future trends [24,25].The BAPC model was implemented using Integrated Nested Laplace Approximations (INLA), which allows for computationally efficient Bayesian inference [26]. Although a log-link function has often been applied in this model, it can lead to extreme estimates. Nordpred addresses criticisms of bayesian models by providing a Bayesian formulation based on the power link, and develops a generalized APC power-link model that assumes the power parameter is random rather than fixed [27]. As a well-established software, Nordpred assumes counts follow a Poisson distribution and incorporates either a log-link function or a power-link function with a fixed power [27].This advanced statistical model, which incorporates historical data alongside probability distributions, facilitated the estimation of future GERD patterns while accounting for the effects of age, period, and cohort. This model has been validated and applied in epidemiological studies globally, particularly in projections of non-communicable disease burdens [28,29].The methodology used adheres to the strengthening the reporting of cohort, cross-sectional, and case–control studies in surgery criteria [30]. All analyses were conducted using R Software (version 4.2.2) and R Studio, with the Nordpred predictive model implemented through the “nordpred (version 1.1)” package.

To validate the stability of the prediction results, we further applied the Nordpred model and Autoregressive Integrated Moving Average (ARIMA) model for cross-validation. ARIMA model is a commonly used time series analysis method [31]. We used this model to predict the prevalence, incidence, and YLDs of GERD in the China from 2022 to 2044. The model can effectively capture the trend and seasonal change characteristics in time series data by integrating three major elements: autoregression, difference, and moving average. ARIMA models analyze time series data to eliminate trends and seasonal effects, revealing underlying long-term patterns while controlling for confounding factors [31]. All analyses were conducted using R Software (version 4.2.2) and R Studio, with the ARIMA model implemented through the “forecast” package.

## Results

### Burden and temporal trend of China in Gastroesophageal Reflux Disease

In China, the prevalent number of GERD cases increased by 60.62% between 1990 and 2021, rising from 50.632 million (35.828–70.009) in 1990 to 81.327 million (57.319–112.992) in 2021 (Table 1). The Age-Standardized Prevalence Rate (ASPR) of GERD was 4067.3 (2876.8–5627.7) per 100,000 population in 1990, which increased to 4827.6 (3407.9–6699.4) per 100,000 in 2021, showing an EAPC of 0.49 (0.37–0.62) (Table 1). Similarly, the number of GERD incidence cases in China reached 32.388 million (21.853–45.181) in 2021, up from 20.863 million (14.218–29.013) in 1990, with the EAPC for the Age-Standardized Incidence Rate (ASIR) increasing by 0.45 (0.33–0.56). The ASIR rose from 1672.6 (1137.8–2327.2) in 1990–1953 (1322.1–2723.1) per 100,000 population in 2021 (Table 1). GERD-related YLDs in China amounted to 0.626 million (0.296–1.118) in 2021, reflecting an increase of 58.88% from 0.394 million (0.185–0.742) in 1990. The Age-Standardized Years of Life Lived with Disability Rate (ASYR) rose from 31.6 (14.8–59.5) per 100,000 population in 1990 to 37.3 (17.6–70.2) in 2021, with an EAPC of 0.48 (0.35–0.61) (Table 1). By 2021, the ASPR, ASIR, and ASYR of GERD in China were approximately half of patient those seen in countries with high SDI (Table 1).

**Table 1:** The numbers and age standardised rate of for incidence, prevalence, and YLDs in Gastroesophageal Reflux Disease in 1990 and 2021 and their temporal trends from 1990 to 2021.

Metrics	location	Number in 1990 (95% CI)	ASR in 1990 (95% CI)	Number in 2021 (95% CI)	ASR in 2021 (95% CI)	EAPC (95% CI)
**Incidence**	**China**	**20863747 (14218142-29012665)**	**1672.6 (1137.8-2327.2)**	**32387865.9 (21852927.5-45181012.8)**	**1953 (1322.1-2723.1)**	**0.45 (0.33-0.56)**
Incidence	Global	180018233.4 (126685177.2-239696589.5)	3375.2 (2375.2-4494.1)	324139599.3 (228740151.2-429198635.5)	4107.5 (2898.6-5438.8)	0.63 (0.61-0.65)
Incidence	High SDI	34136778.5 (23677541.4-46183084.2)	3072.4 (2134-4162.3)	47166281.1 (32596273.9-63956297.2)	3562.2 (2465.3-4836)	0.42 (0.36-0.48)
Incidence	High-middle SDI	35652655.2 (24873483.3-48083966.6)	2942.9 (2051.8-3977.1)	50357993.3 (35317090.5-67692995)	3314.3 (2328-4465.6)	0.34 (0.28-0.4)
Incidence	Low SDI	16607901.8 (11694710.5-22121058.1)	4335.2 (3058.4-5746.6)	39536248.6 (27820713.9-52726535.3)	5113.7 (3608.7-6748.3)	0.55 (0.54-0.57)
Incidence	Low-middle SDI	43396197.6 (30812886.2-57161430.8)	4397.6 (3129.1-5767.2)	88397917.4 (62892937.2-116058491.5)	5194.4 (3702.5-6778)	0.55 (0.53-0.57)
Incidence	Middle SDI	50019121.9 (35396569-66322065.8)	3030.5 (2145.1-4008.6)	98388205.6 (69843407.1-129271460.8)	3866.8 (2745.1-5083.9)	0.8 (0.78-0.81)
**Prevalence**	**China**	**50632181.1 (35828617.1-70009490.6)**	**4067.3 (2876.8-5627.7)**	**81327260.3 (57319226-112992013.9)**	**4827.6 (3407.9-6699.4)**	**0.49 (0.37-0.62)**
Prevalence	Global	450765454.7 (328865601.3-595474409.5)	8451.4 (6165.9-11164.5)	825603654.1 (604057909.5-1084812340.4)	10462.1 (7654.7-13746.8)	0.67 (0.65-0.7)
Prevalence	High SDI	87040987.1 (63076306.6-116773248.8)	7646 (5534.6-10257.8)	120765088.8 (86974132-162749378.7)	8939.9 (6423.6-12054.7)	0.43 (0.35-0.5)
Prevalence	High-middle SDI	90575890.7 (65635343.9-121068965.6)	7404.3 (5360.7-9906)	131003975.1 (96064951.5-173582153.6)	8475.6 (6215.2-11242.5)	0.38 (0.32-0.45)
Prevalence	Low SDI	40756198.7 (29609362.7-53834601.1)	10863.6 (7907.7-14318)	96970987.2 (70418904.8-128192254.8)	13097.4 (9551.8-17221.2)	0.62 (0.61-0.63)
Prevalence	Low-middle SDI	107823563.6 (79002199.8-141021584.7)	11093.5 (8146.3-14476.6)	223640427.3 (164034919.3-291809031.4)	13402.5 (9858.9-17425.1)	0.61 (0.6-0.63)
Prevalence	Middle SDI	124042192 (90688353.9-163014433.2)	7599.6 (5561.8-9976.5)	252457133.8 (185150464.2-329834437.9)	9889.3 (7256-12920.6)	0.86 (0.84-0.87)
**YLDs**	**China**	**393857.5 (185082.4-741986)**	**31.6 (14.8-59.5)**	**626247.5 (295851.6-1177532.1)**	**37.3 (17.6-70.2)**	**0.48 (0.35-0.61)**
YLDs	Global	3472702.6 (1664265.6-6450647.2)	65.1 (31.2-120.9)	6336161.7 (3050659.6-11745436.2)	80.3 (38.7-148.8)	0.66 (0.64-0.69)
YLDs	High SDI	668088.1 (320651.2-1241480.5)	59 (28.2-110.1)	919090.8 (441430.9-1710242.6)	68.6 (32.8-128.2)	0.41 (0.34-0.49)
YLDs	High-middle SDI	697106.4 (332849.7-1290484.1)	57.1 (27.2-105.9)	1003165.1 (485189.9-1848249.7)	65.2 (31.4-120.3)	0.38 (0.31-0.45)
YLDs	Low SDI	313170.2 (149645.6-581822.2)	83.1 (39.8-154.2)	748244.5 (356320.1-1392393.5)	100.1 (48-185.6)	0.62 (0.61-0.64)
YLDs	Low-middle SDI	829855.4 (399391.4-1536723)	85.1 (41-157.2)	1718835.4 (828533.9-3177009.4)	102.4 (49.6-188.7)	0.6 (0.59-0.62)
YLDs	Middle SDI	960429.5 (460232.5-1784680)	58.7 (28.2-108.9)	1940951.3 (935810.4-3597074.3)	76 (36.6-140.7)	0.84 (0.82-0.86)

Abbreviations: APC, Annual Percentage Change; ASR, Age-Standardized Rate; EAPC, Estimated Annual Percentage Changes; SDI, Socio-Demographic Index; YLDs, Years of Life Lived with Disability.

**Fig 1:**
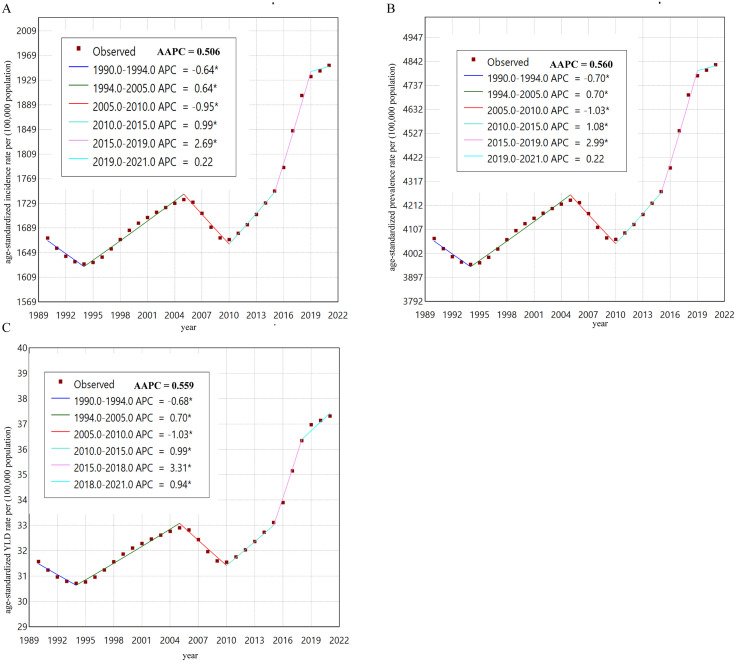
The joinpoint regression analysis model for Gastroesophageal Reflux Disease in China from 1990 to 2021. (A) The joinpoint regression analysis model of ASR for incidence. (B) The joinpoint regression analysis model of ASR for prevalence. (C) The joinpoint regression analysis model of ASR for YLDs. Abbreviations: APC, Annual Percentage Change; AAPC, Average Annual Percent Change; ASR, Age-Standardized Rate; YLDs, Years of Life Lived with Disability.

The detailed analysis, utilizing both APC and AAPC, revealed differing trends. Prior to 2010, GERD prevalence, incidence, and YLD rates in China exhibited only slight variations (Fig 1). However, following this period, from 2010 to 2021, there was a marked and substantial increase in these rates (Fig 1). Over the past three decades, the overall AAPC for the prevalence rate was 0.56%, for incidence it was 0.51%, and for YLDs it was 56%, all indicating an upward trend exceeding 50% (Table 2 and Fig 1).

**Table 2:** The numbers and age standardised rate of for incidence, prevalence, and YLDs in Gastroesophageal Reflux Disease of China in 1990 and 2021,along with the relative changes and AAPC in ASR per 100 000 cases from 1990 to 2021.

Location	Metrics	Number in 1990 (95% CI)	ASR in 1990 (95% CI)	Number in 2021 (95% CI)	ASR in 2021 (95% CI)	Relative change of numbers from 1990 to 2021 (%)	Relative change of ASR from 1990 to 2021 (%)	AAPC	P-Value
China	Incidence	20863747 (14218142-29012665)	1672.6 (1137.8-2327.2)	32387865.9 (21852927.5-45181012.8)	1953 (1322.1-2723.1)	55.24	16.76	**0.51(0.43-0.58)**	**<0.001**
China	Prevalence	50632181.1 (35828617.1-70009490.6)	4067.3 (2876.8-5627.7)	81327260.3 (57319226-112992013.9)	4827.6 (3407.9-6699.4)	60.62	18.69	**0.56(0.47-0.65)**	**<0.001**
China	YLDs	393857.5 (185082.4-741986)	31.6 (14.8-59.5)	626247.5 (295851.6-1177532.1)	37.3 (17.6-70.2)	59.00	18.04	**0.56(0.46-0.66)**	**<0.001**

Abbreviations: APC, Annual Percentage Change; AAPC, Average Annual Percent Change; ASR, Age-Standardized Rate; YLDs, Years of Life Lived with Disability.

### Trend of China in Gastroesophageal Reflux Disease by sex and age group

**Fig 2:**
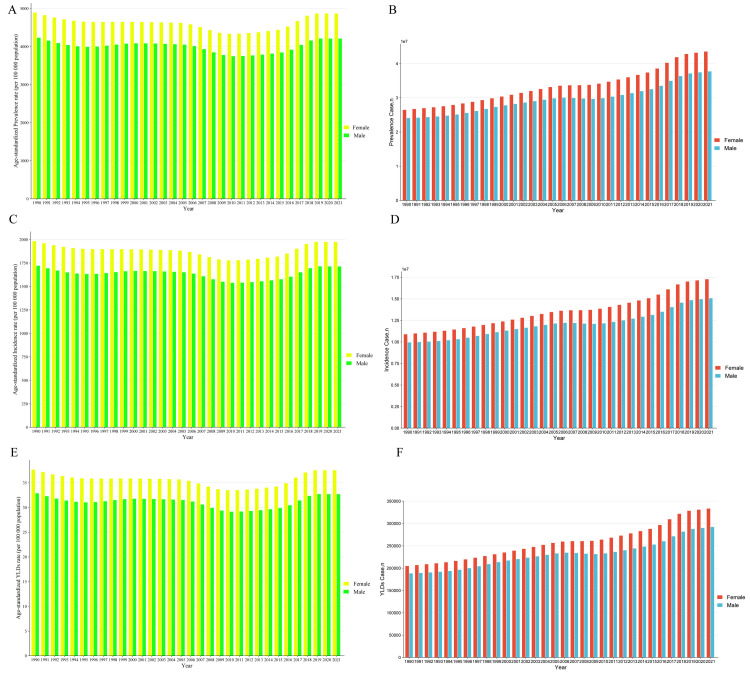
The trends in ASR and number of prevalence (A,B), incidence (C,D), and YLDs (E,F) for Gastroesophageal Reflux Disease of China among different sexes, female and male, from 1990 to 2021. Abbreviations: ASR, Age-Standardized Rate; YLDs, Years of Life Lived with Disability.

Over the course of the past three decades, the ASRs for both males and females show noticeable fluctuations. While the fluctuation trends for both sexes are similar, females consistently show higher rates across all categories (Fig 2A, 2C and 2E). In contrast, when considering absolute numbers, a clear upward trend emerges, especially in the areas of prevalence, incidence, and YLDs (Fig 2B, 2D and 2F).

**Fig 3:**
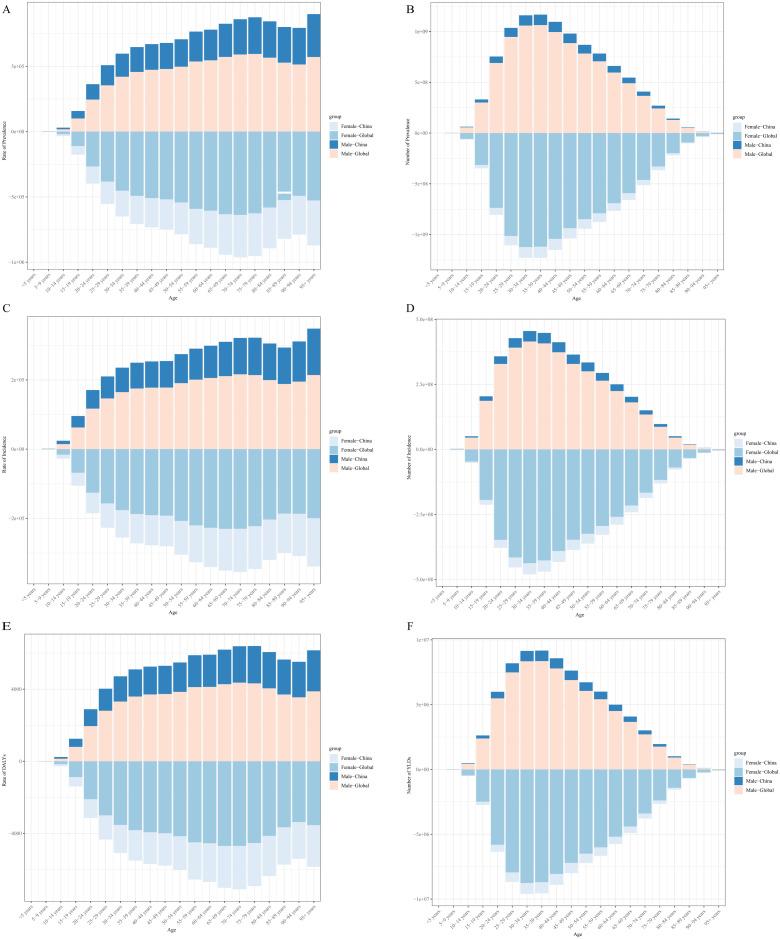
The trends in rate and number of prevalence (A,B), incidence (C,D), and YLDs (E,F) for Gastroesophageal Reflux Disease of China among different age in 2021. Abbreviations: YLDs, Years of Life Lived with Disability.

Both prevalence, incidence, and YLDs rates increase with age, reaching their highest levels in the 70–74 year age group. Females continue to exhibit higher rates compared to males across these metrics (Fig 3A, 3C and 3E). However, when looking at the absolute numbers, a distinct peak is observed in the 30–39 year age group for all these metrics (Fig 3B, 3D and 3F).

### Frontier analysis

**Fig 4:**
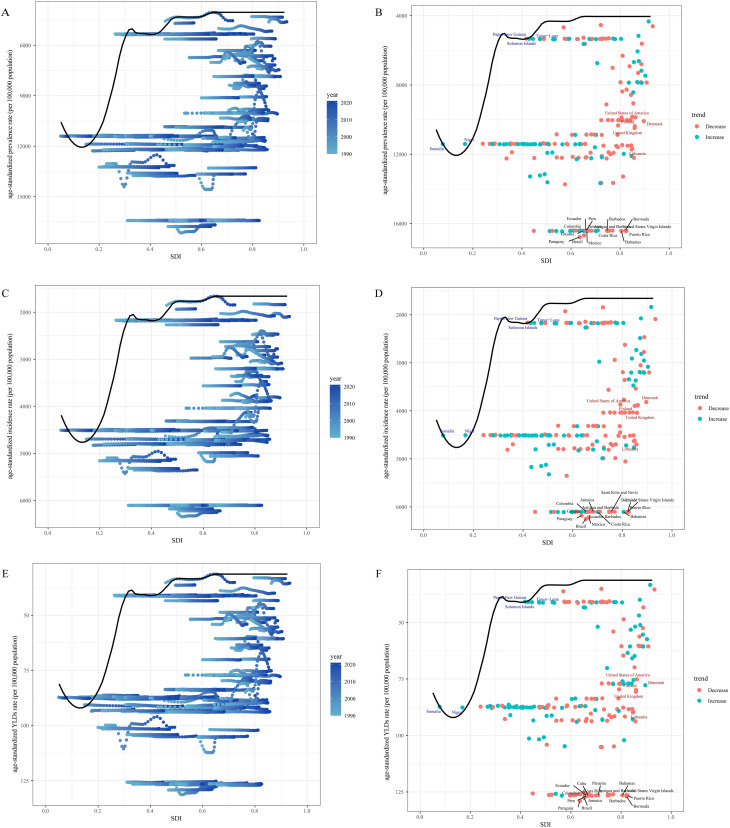
Frontier analysis of the relationship between the Socio-Demographic Index (SDI) and Age-Standardized Rate (ASR) of gastroesophageal reflux disease (GERD). (A,C, and E) The effective difference from the frontier for each country or territory by single year (2021 vs. 1990). (B, D, and F) The effective difference from the frontier for each country or territory in 2021.

In the comprehensive frontier analysis, which examined the SDI and ASRs of GERD from 1990 to 2021 across 204 countries and territories, distinct patterns were identified. As the SDI increased from 0.0 to 1.0, the Age-Standardized Rates for GERD prevalence generally decreased, reflecting an overall reduction in the disease burden. A similar trend was observed for the incidence rate of GERD, which also showed a decline with rising SDI. Likewise, the YLDs followed a comparable pattern, suggesting that as socio-economic development progresses, the burden of GERD tends to lessen (Fig 4A, 4C and 4E).

The frontier analysis conducted in 2021 highlighted distinct trends in the GERD burden across various countries and territories. Notably, 15 countries, including the Bahamas, Puerto Rico, and Bermuda, were found to have significantly higher rates of GERD prevalence and incidence, positioning them far from the frontier. These countries showed a considerable gap between their observed burden and what would be expected based on their SDI. In contrast, nations like Timor-Leste, Papua New Guinea, and the Solomon Islands were observed to be positioned closer to the frontier, reflecting more optimal outcomes relative to their SDI values. Despite their higher SDI, countries such as Denmark, the United Kingdom, and Lithuania were found to have substantial effective differences, indicating that their GERD burden did not align with what would be expected based on their developmental stages (Fig 4B, 4D and 4F). According to the results of this analysis, China displayed relatively lower prevalence and incidence rates, coupled with a high-middle SDI value of 0.7216, positioning it near the frontier (S1 Table).

### Cross-country inequality analysis

**Fig 5:**
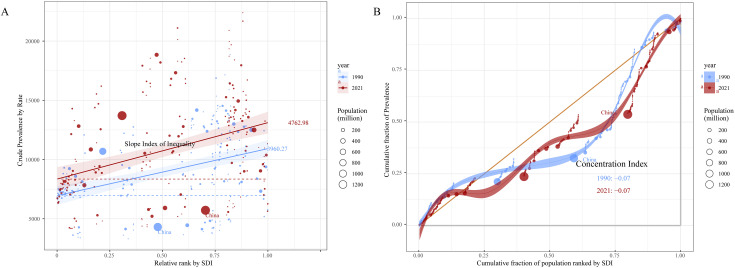
Health inequality regression curves (A) and concentration curves (B) for the prevalence of Gastroesophageal Reflux Disease from 1990 to 2021 across the world.

In 1990 and 2021, the SII (per 100,000 population) for GERD prevalence were 3960.27 (95% CI 2470.80 to 5449.73) and 4762.98 (95% CI 2737.34 to 6788.62), respectively, reflecting a disproportionate burden in countries with higher SDI (Fig 5A). The analysis revealed both absolute and relative inequalities related to SDI, with a noticeable increase in the inequality of the age-standardized GERD burden from 1990 to 2021 (Fig 5A). The CI for prevalence exhibited a same trend between 1990 and 2021I (Fig 5B). Additionally, a significantly greater inequality in GERD burden was observed in China compared to other countries, positioning the country further from the diagonal line, suggesting a disparity in GERD distribution relative to SDI (Fig 5B).

### Trends of Gastroesophageal Reflux Disease from 1990 to 2044

**Fig 6:**
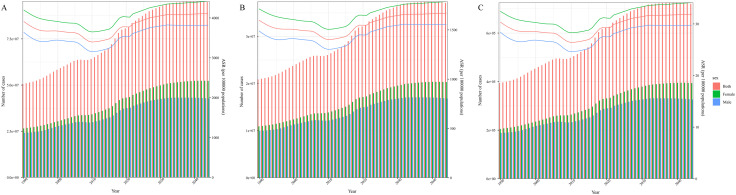
Projected ASR and numbers of prevalence (A), incidence (B), and YLDs (C) for Gastroesophageal Reflux Disease by gender (both, male, and female) from 1990 to 2044 based on the Nordpred models. Curve signifies the trend of ASR. Abbreviations: ASR, Age-Standardized Rate; YLDs, Years of Life Lived with Disability.

Using the Nordpred models, we have projected the trend of GERD for both sexes from 2022 to 2044 (S2 Table). The trends in the combined numbers for prevalence, incidence, and YLDs for both sexes from 1990 to 2044 show a steady upward trajectory. Specifically, the number of cases for both prevalence and incidence has increased consistently among females. For males, however, the numbers rise until 2035 and then gradually decrease thereafter. The ASPR, ASIR, and ASYR for both sexes initially decline until 2010, followed by a sharp rise from 2010 to 2035, after which the rates stabilize through 2044. Both male and female ASRs follow this general trend, aligning with each other over the forecast period (Fig 6).

**Fig 7:**
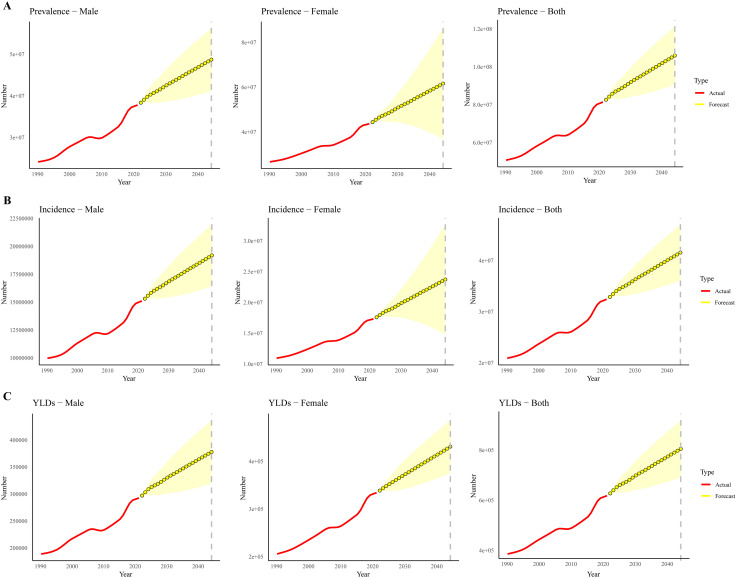
Projected numbers of prevalence (A), incidence (B), and YLDs (C) for Gastroesophageal Reflux Disease by gender from 1990 to 2044 based on the ARIMA models. Abbreviations: ASR, Age-Standardized Rate; YLDs, Years of Life Lived with Disability.

To avoid the potential limitations of relying on a single model, we have supplemented the predictive analysis with a complementary validation using the ARIMA model. From the ARIMA models, we find that the trends in the combined numbers for prevalence, incidence, and YLDs for both sexes from 2022 to 2044 show a steady upward trajectory (S3 Table). Specifically, the numbers of cases for prevalence, incidence, and YLDs have increased consistently among males and females (Fig. 7). The ARIMA models further confirmed the overall trends in prevalence, incidence, and YLDs.

## Discussion

Between 1990 and 2021, the number of GERD prevalence significantly increased by 60.62%, reaching 81.327 million, and the ASPRs rose to 4827.6 (3407.9–6699.4) per 100,000 population. The total number of incident cases increased by 55.24%, with the ASIR reaching 1953 (1322.1–2723.1) per 100,000 population in 2021. GERD-related YLDs also saw a marked increase of 58.88%, with the ASYR rising by 18.03%. Females consistently exhibited higher rates across all metrics, and both sexes experienced substantial increases over time, particularly in terms of absolute numbers. Among age groups, elderly individuals, especially those between 70 and 74 years of age, showed the highest rates for prevalence, incidence, and YLDs. Projections from 2022 to 2044 indicate a continuous rise in the combined prevalence, incidence, and YLDs for both sexes.

Between 1990 and 2021, both male and female populations in China experienced an increase in the ASRs and total number of GERD cases, reflecting a significant rise in the overall GERD burden. This trend aligns with findings from previous domestic Chinese studies focusing on the prevalence of gastroesophageal reflux disease [13]. The observed rise in GERD cases is believed to be strongly associated with the growing obesity epidemic, which has been shown to increase the risk of GERD by up to three times [32]. Additionally, the decline in the prevalence of Helicobacter pylori-associated gastritis may have played a contributing role in this upward trend [7]. When researchers studied GERD in five Chinese regions, they discovered it affected between 1.7% and 5.1% of people, with Shanghai having the most cases at 6.4% [33,34]. It is important to note that certain earlier studies [9] employed relatively broader diagnostic criteria. In contrast, the GERD definition used in the GBD 2021 Study is based on expert recommendations [35], requiring symptoms to occur at least once a week for a duration of 12 months, thus mitigating the potential for overestimation. Our study shows that GERD becomes more common as people get older, and women get it more than men, matching findings from the GBD 2019 research [12]. The phenomenon that the burden of GERD is associated with sex differences may be related to estrogen levels. Previous studies have indicated that menopause is a risk factor for GERD, which is linked to decreased levels of estrogen and progesterone [36]. Furthermore, studies have revealed a positive association between the occurrence of gastroesophageal reflux and patients with bipolar disorder [37]. Notably, elevated fluctuations in estrogen levels may enhance the regulation of inflammatory processes within the central nervous system, which contributes to an increased incidence of bipolar disorder in women and, consequently, a higher risk of GERD [37]. Furthermore, Our study observed that the EAPC confirms a steady long-term increase in the GERD burden, while the AAPC reveal that this growth accelerated after 2010—an trend that may be associated with changes in China’s socioeconomic status and healthcare system during that period. On one hand, as China became the world’s second-largest economy in 2010, the growth in residents’ income boosted their health awareness and increased the proportion of household consumption allocated to healthcare. On the other hand, the launch of China’s healthcare system reform in 2009 and the expansion of basic medical insurance coverage reduced financial barriers to endoscopy and physician consultations, enabling more timely identification of symptomatic cases, encouraging more people to proactively participate in health check-ups and disease screenings—thereby increasing the detection rate of GERD.

When looking at 204 countries from 1990 to 2021, the frontier analysis shows that GERD rates go down as countries improve their SDI scores, showing the phenomenon that better SDI results are important for creating good health policies. The results point out major variations among the examined nations. Even though Bermuda has a high SDI score, it shows much higher GERD rates both on prevalence and incidence levels than expected, which puts it far from reaching optimal health standards. Despite being a high-middle SDI nation, China reports lower numbers of people with GERD and new cases each year. The difference between countries shows that healthcare results are complex – even though SDI is important, things like environment, genetics, and how healthcare systems work also matter for GERD patterns [38]. Using SDI data, the SII and CI values were calculated in our study to track differences of inequality. The data shows that inequality increased in 2021 when we look at SII numbers, yet the Concentration Index didn’t show much change. Furthermore, the inequality analysis reveals significant disparities of inequality in GERD burden within China.

This study has several strengths and limitations. One of the main challenges is the potential variability in data accuracy and consistency across different regions, which can introduce biases or inaccuracies. Studies conducted in different areas within the same region may reflect cultural, ethnic, and geographic differences, as noted in previous research [39]. Differences in study design and data collection methods can affect the precision of the estimates. As previously discussed, diagnostic criteria play a crucial role in influencing the results [35]. Future studies should aim to be more geographically and demographically consistent to reduce such disparities. Furthermore, while our projections extend to 2044 and are based on robust statistical models, which may be affected by uncertain assumptions in the future, including the potential influence of evolving diagnostic standards and advancements in medical technology over time. Additionally, Our study is based on summary data from the GBD Study—national-level estimates—which may mask subnational variations across China’s diverse provinces. To date, there has been no large-scale, nationally representative population-based survey specifically focused on GERD at the provincial level in China. While we cannot address this gap due to objective data constraints, our study uses the most reliable data available (GBD national-level estimates) to ensure scientific rigor. While provincial-level data would be ideal for local policy, the national-level GERD burden findings presented in our study remain highly meaningful for public health practice, and these findings cannot be fully substituted for provincial-level data. China’s public health policies (e.g., the “Healthy China 2030” Initiative) first require evidence-based national-level targets. This study establishes a comprehensive 32-year (1990–2021) baseline of China’s GERD burden, including long-term prevalence trends and age-sex disparities. This baseline acts as a “north star” for provincial policymakers: once local data is available, provinces can benchmark their local GERD trends against the national average to identify gaps, rather than developing interventions without a national reference. By synthesizing national GERD burden characteristics (e.g., age/sex-specific trends), the study identifies clear “priority populations” and “high-burden trends” to guide future provincial surveys. For example, its finding that GERD prevalence grows fastest in a specific age group suggests provincial studies can prioritize this group, avoiding unfocused large-scale surveys and improving research efficiency.

To the best of our knowledge, this is the most recent estimate of GERD prevalence in the Chinese population. Compared with the previous study of GBD 2019 [11,12], this study utilized the GBD 2021 database, which supplements data on the changes in disease burden during the COVID-19 pandemic (2020–2021). The COVID-19 pandemic has exerted an impact on people’s lifestyles, and these data reflect the influence of short-term social factors on the burden of GERD. In fact, high-quality epidemiological studies on GERD in China have been scarce, and, to date, no large-scale, ethnicity-inclusive national survey on GERD has been conducted. Previous global burden of GERD studies based on the GBD database prioritized the exploration of global or continental patterns in disease burden distribution. In these studies, data from China were only incorporated into global statistical tables in the form of “national-level total prevalence and incidence rates” [11,12].The temporal dynamic trends of China’s disease burden have not been analyzed separately. Moreover, previous global burden studies on GERD have only analyzed GERD in China at the “overall population” level, without leveraging the refined annotations of the GBD database [11,12]. For the first time, this study decomposes the temporal dynamic trends of GERD burden in China based on GBD data, conducting an in-depth analysis with “sex stratification plus age disaggregation” to identify patterns that have not been previously reported. The GBD 2021 Study, which included a broader range of studies, addressed the variability among previous studies, providing the most current estimates of GERD’s epidemiology. This analysis examined the trend over time and the negative impact of GERD on patients’ quality of life, using AAPC and YLDs. These findings may serve as valuable references for researchers and policymakers in preparing clinicians and healthcare systems to prioritize management strategies in the future. The “Healthy China 2030” Planning Outline Initiative, which establishes a long-term strategic framework (2016–2030), takes 2030 as a crucial phased endpoint and clarifies the core development goals that China needs to achieve in the health sector by 2030. Our projection to 2044 extends this timeline to provide evidence for post-2030 policy formulation, addressing the need for long-term foresight in chronic disease control. Given the significant impact of GERD on patients’ quality of life and its association with an increased risk of esophageal adenocarcinoma [4], there is a clear need for continuous monitoring and adaptive health strategies in China.

In conclusion, data from the last thirty years shows China’s GERD disease burden keeps increasing demonstrated by higher ASPR, ASIR, and ASYR values. The increasing numbers show the need of focusing on health differences of inequality and social factors affecting GERD throughout China. The rising GERD rates mean healthcare approaches need to update while tracking disease progression at all times. Healthcare systems need to enhance their capabilities because the rising number of GERD patients requires more medical services. To reduce the burden of GERD in the future, raising awareness, particularly regarding early diagnosis and treatment, is essential. Special attention should be given to individuals with risk factors to ensure that GERD is managed proactively. Additionally, future research into GERD’s epidemiology in the Chinese population must be strengthened, as better data will provide insights necessary for improving public health strategies and care management.

## Supporting information

S1 Table Details of result of Frontier analysis. (XLSX)

S2 Table Details of result of Nordpred models. (XLSX)

S3 Table Details of result of ARIMA models. (XLSX)

S1 Data Minimal dataset for the study. This compressed ZIP archive contains all underlying data required to reproduce the figures and tables presented in the manuscript. These supporting information has been uploaded to a public repository and is accessible via the following https://doi.org/10.6084/m9.figshare.30287137.
(ZIP)

## Abbreviations

AAPC: Average Annual Percentage Change
APC: Annual Percentage Change
ARIMA: Autoregressive Integrated Moving Average
ASIR: Age-Standardized Incidence Rate
ASPR: Age-Standardized Prevalence Rate
ASR: Age-Standardized Rate
ASYR: Age-Standardized Years of Life Lived with Disability Rate
BAPC: Bayesian Age-Period-Cohort
CI: Concentration Index
EAPCs: Estimated Annual Percentage Changes
GBD: global burden of Disease
GERD: Gastroesophageal Reflux Disease
GHDx: Global Health Data Exchange
SDI: Socio-Demographic Index
SII: Slope Index of Inequality
YLDs: Years of Life Lived with Disability.
